# Cost Analysis of PSMA-PET in the PROSPET-BX Trial

**DOI:** 10.3390/cancers18050806

**Published:** 2026-03-02

**Authors:** Egesta Lopci, Cesare Saitta, Alberto Saita, Elena Vanni, Alessandro Santandrea, Luca Disconzi, Vittorio Fasulo, Nicolò Buffi, Massimo Lazzeri, Giovanni Lughezzani

**Affiliations:** 1 Nuclear-Medicine Unit, IRCCS—Humanitas Research Hospital, 20089 Rozzano, MI, Italy; 2 Urology Unit, IRCCS—Humanitas Research Hospital, 20089 Rozzano, MI, Italy; 3 Department of Biomedical Sciences, Humanitas University, 20072 Pieve Emanuele, MI, Italy; 4 Business Operating Office, IRCCS—Humanitas Research Hospital, 20089 Rozzano, MI, Italy; 5 Radiology Department, IRCCS—Humanitas Research Hospital, 20089 Rozzano, MI, Italy

**Keywords:** [^68^Ga]PSMA-11, PSMA-PET, PET/CT, prostate cancer, mpMRI, cost-effectiveness, PROSPET-BX

## Abstract

Background: The PROSPET-BX trial compared [^68^Ga]PSMA-11 PET/CT (PSMA-PET) with multiparametric MRI (mpMRI) in parallel in men with suspicion of prostate cancer (PCa) after at least one previously negative biopsy (ClinicalTrials.gov: NCT05297162; GR-2018-12366240). In this study, we performed the cost analysis of the two imaging modalities with respect to the detection of clinically significant PCa (csPCa). Methods: We analyzed the data from patients enrolled in the trial who met the inclusion criteria. For the cost analysis, we identified six competing triage strategies, each defined as a binary decision rule for referral to prostate biopsy: (1) biopsy-all; (2) elevated PSA-density (PSAD; biopsy if PSAD > 0.15 ng/mL/cc; (3) mpMRI positive (PIRADS 3–5); (4) PSMA-PET positive (PRIMARY 3–5); (5) mpMRI or PSMA-PET positive; (6) PSAD and mpMRI. For each strategy, we yielded sensitivity, specificity, positive predictive value (PPV), negative predictive value (NPV), and accuracy for csPCa. Direct hospital costs were modeled from a provider perspective, incorporating testing and procedural costs. Unit costs (in EUR) were sourced from our institutional accounting records. Pairwise cost-effectiveness comparisons were performed using incremental cost-effectiveness ratio (ICER) and incremental net benefit (INB). Results: Among the six triage strategies evaluated, the “biopsy-all” approach achieved perfect sensitivity, whereas the PSAD + mpMRI pathway was the most parsimonious strategy but missed 14 csPCa cases (53.8%). The combined “mpMRI or PSMA-PET” strategy maximized detection (22 cPCa, missing only 4) at an intermediate cost (EUR 81.991 total; EUR 3.727 per csPCa). The pairwise comparison of each strategy with mpMRI alone showed for the mpMRI or PSMA-PET pathway a low ICER (~EUR 2.900/extra csPCa), with consistently positive and increasing INB across higher WTP (willingness-to-pay). Therefore, this combination provided the most favorable cost-effectiveness profile, balancing detection, efficiency, and cost. Conclusions: To the best of our knowledge, this is the first cost analysis study to compare different strategies incorporating PSMA-PET in the re-biopsy setting, demonstrating that the combined “mpMRI or PSMA-PET” pathway is the most cost-effective diagnostic pathway for csPCa detection.

## Simple Summary

This first re-biopsy cost analysis study, derived from the PROSPET-BX trial (NCT05297162), highlights combined imaging as the optimal strategy for clinically significant prostate cancer (csPCa) detection. More specifically, analyses of six triage strategies showed that the combination of mpMRI or PSMA-PET positive was most cost-effective, detecting the majority of csPCa (22/26) with a low incremental cost-effectiveness ratio (ICER) (~EUR 2900/extra case) versus mpMRI alone, positive incremental net benefit (INB) across willingness-to-pay (WTP) thresholds, and balanced efficiency.

## 1. Introduction

The core objective of image-guided biopsy for prostate cancer detection is to accurately differentiate men with and without cancer, reliably identify clinically significant disease, and efficiently assess tumor burden to guide treatment decisions. Multiparametric MRI (mpMRI) has become the preferred imaging method for prostate cancer (PCa) detection and is now incorporated into national and international guidelines [1,2]. However, mpMRI has notable limitations, and even with standardized Prostate Imaging Reporting and Data System (PI-RADS) versions 1 and 2, it can yield equivocal results and occasionally miss clinically significant prostate cancer (csPCa) [3].

In this setting, PSMA (prostate-specific membrane antigen) PET/CT (PSMA-PET) emerges as a highly promising alternative to mpMRI. As a whole-body imaging tool, it enables comprehensive disease evaluation by detecting and staging tumors simultaneously. In our initial exploratory work, we demonstrated the technical feasibility of integrating PET/TRUS fusion-guided biopsy [4], followed by a prospective study assessing PSMA-PET’s effectiveness for primary PCa detection in the same cohort of patients who were mpMRI-negative or had contraindications to mpMRI [5]. Using optimal PSMA thresholds, we achieved 90% accuracy for csPCa detection. Compared to mpMRI, PSMA-PET demonstrated 84% accuracy and 97% specificity [6]. Notably, PSMA PET/CT identified clinically significant tumors (GS  ≥  7) missed by mpMRI in 25% of cases. Similar favorable results were seen when PSMA PET/CT was compared to micro-ultrasound imaging [7].

The prospective trial PROSPET-BX (ClinicalTrials.gov: NCT05297162) was designed to show that [^68^Ga]PSMA-11 PET/CT (“experimental test”) provides superior diagnostic performance compared to mpMRI (“standard test”) in patients with high suspicion of PCa after a prior negative biopsy [8]. As a secondary aim, we sought to evaluate the clinical utility of the “experimental test” versus the “standard test”, with a focus on cost analyses. This study specifically reports the cost analyses from the PROSPET-BX trial across various scenarios.

## 2. Materials and Methods

### 2.1. Study Population

The PROSPET-BX trial compares in parallel [^68^Ga]PSMA-11 PET/CT (or PSMA-PET) (“experimental test”) with mpMRI (“standard test”) fusion-guided prostate biopsy in men with clinical and laboratory suspicion of PCa following at least one prior negative biopsy. The primary clinical endpoint was detection of clinically significant prostate cancer (csPCa), defined as ISUP (International Society of Urological Pathology) grade group ≥ 2 (cs) [9]. This analysis evaluates the clinical utility of the “experimental test” relative to the “standard test”, emphasizing procedural impact and cost-effectiveness. Inclusion criteria, as previously described [8], included the following: adults (age > 18 years); PSA level > 4.0 ng/mL; free-to-total PSA ratio < 20%; rising PSA on two consecutive measures; ≥1 prior negative biopsy or high-grade prostatic intraepithelial neoplasia (HG-PIN)/atypical small acinar proliferation (ASAP); and negative digital rectal exam. The study was approved by the Ethics Committee of the IRCCS Humanitas Research Hospital (ID 3131) and registered at ClinicalTrials.gov (NCT05297162). All participants provided written informed consent prior to enrolment. Patients underwent both PSMA-PET and mpMRI before repeat biopsy. Detailed information on imaging protocols has been previously reported [8].

### 2.2. Diagnostic Strategies

**Figure 1:**
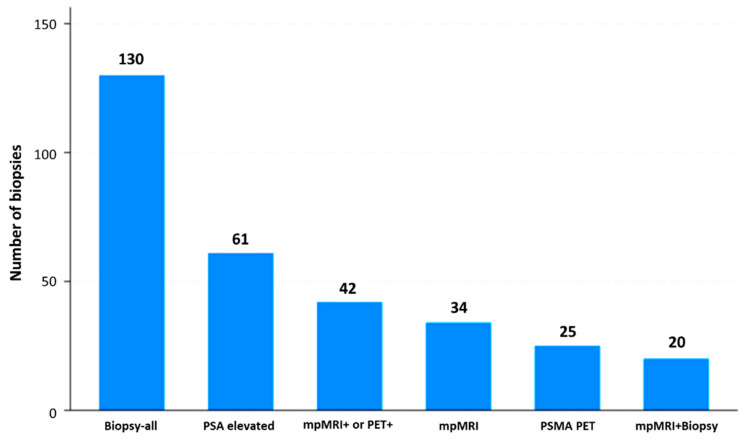
Distribution of prostate fusion biopsies with respect to the six triage strategies.

For the cost analysis, we defined six competing triage strategies, each as a binary rule for prostate biopsy referral (Figure 1):

1. **Biopsy-all:** Biopsy all men regardless of clinical and imaging results.
2. **PSA-density elevated:** Biopsy if prostate-specific antigen density (PSAD) > 0.15 ng/mL/cc.
3. **mpMRI positive:** Biopsy if mpMRI shows PIRADS 3–5 lesions [10].
4. **PSMA-PET positive:** Biopsy if PSMA-PET shows PRIMARY 3–5 lesions [11].
5. **mpMRI or PSMA-PET:** Biopsy if either mpMRI (PIRADS ≥ 3) or PSMA-PET is positive.
6. **PSAD and mpMRI:** Biopsy only if both PSAD > 0.15 ng/mL/cc and mpMRI are positive.

For each strategy, we generated 2 × 2 contingency tables against the csPCa reference standard to calculate sensitivity, specificity, positive predictive value (PPV), negative predictive value (NPV), and accuracy. Number needed to biopsy (NNT) was calculated as biopsies/csPCa detected. Missed csPCa (false negatives) were counted relative to the biopsy-all strategy. For each procedure, we referred to the reimbursement costs provided by the Business Operating Officers of our institution according to the Italian National Health System. Table 1 illustrates the specific costs per single procedure.

### 2.3. Costing Framework

Direct hospital costs were modeled from a provider perspective, incorporating both testing and procedural costs. Unit costs (in EUR) were sourced from our institutional accounting record.

Two cost-allocation paradigms were applied:

- ***Stepwise allocation:***
  − In mpMRI or PSMA-PET, all patients undergo mpMRI, and PET is performed only in those with negative mpMRI but positive PET findings.
  − In PSA and mpMRI, all patients undergo PSA, with mpMRI performed only in those with elevated PSA.
- ***Universal allocation (sensitivity analysis):*** all tests specified by a strategy are performed in all patients, irrespective of upstream results.

Furthermore, pairwise cost-effectiveness comparisons were performed using the Stata18.5/SE (StataCorp, College Station, TX 77845 USA) command heabs, which computes the incremental cost-effectiveness ratio (ICER) and the incremental net benefit (INB) for a new strategy versus a comparator. A positive INB indicates that the new strategy is cost-effective relative to the comparator at the specified WTP (willingness-to-pay) threshold. We implemented a custom wrapper to reformat diagnostic strategies into a two-arm trial structure, enabling bootstrap-based ICER and INB estimation for each pairwise comparison. ICERs and INBs were estimated for multiple WTP thresholds (EUR 5000, EUR 10,000, and EUR 20,000 per csPCa detected). Incremental cost-effectiveness was captured by the cost per csPCa detected (total cost/detected). Confidence intervals (CIs) for ICERs and INBs are derived using the Fieller method for ratios and delta method for net benefits, which is standard in cost-effectiveness analysis. ICER = ΔCost/ΔcsPCa; 95% CI via Fieller’s theorem accounting for covariance between cost and effect variances. INB = (ΔcsPCa × WTP) − ΔCost; 95% CI = estimate ± 1.96 × SE_INB, where SE_INB ≈ sqrt(SE_ΔCost^2^ + (WTP × SE_ΔcsPCa)^2^ + 2 × WTP × cov(ΔC,ΔE)). SEs estimated from binomial variance for csPCa (*n* = 130, *p* = 26/130 = 0.2) and ~15–20% CV for costs (typical in diagnostic studies) [12].

The dataset was analyzed using Stata18.5/SE package software (StataCorp, College Station, TX 77845 USA) and in a python environment.

### 2.4. Literature Review

The literature review of cost analysis studies was conducted on MEDLINE, PubMed, and EMBASE databases for published articles until October 2025. The search string included ((PSMA[Text Word]) OR (prostate-specific membrane antigen[Text Word])) AND ((positron emission tomography[Text Word]) OR (PET[Text Word])) AND ((prostate cancer[Text Word]) OR (clinically significant prostate cancer [Text Word]) OR (csPCa[Text Word])) AND ((prostate biopsy[Text Word]) OR (fusion biopsy[Text Word])) AND ((mpMRI[Text Word]) OR (multiparametric MRI[Text Word])) AND ((cost-effectiveness[Text Word]) OR (cost utility[Text Word]) OR (cost analyses[Text Word])). We excluded paper in other languages and not pertinent to the topic.

## 3. Results

### 3.1. Cost Analyses in the Study Cohort

Overall, 130 patients were analyzed [13]. Table 2 shows the cost-effective analysis strategies analyzed. Among the six triage strategies evaluated, the biopsy-all approach achieved perfect sensitivity, identifying all 26 clinically significant (cs) cancers, but at the cost of subjecting every patient to biopsy and yielding the highest number needed to test (NNT = 5.0) and the greatest resource burden (EUR 116.058 total; EUR 4.464 per cs cancer detected). On the other hand, the PSA-density-based strategy (PSAD > 0.15) reduced the number of biopsies by more than half (61/130) while still detecting 17 csPCa. This corresponded to a favorable cost-per-detection ratio (EUR 3.210 per cs cancer), albeit at the expense of missing nine cs cases. In a similar manner, mpMRI triage (PI-RADS 3–5) achieved the same detection yield (17 cs cancers) with even fewer biopsies (34/130), improving diagnostic efficiency (NNT = 2.0). However, total costs remained higher due to imaging (EUR 67.357 total; EUR 3.962 per cs cancer). The PSMA-PET–based strategy detected 18 cs cancers with only 25 biopsies (NNT = 1.4) but incurred markedly higher diagnostic costs, translating into the least favorable cost-effectiveness profile (EUR 8.023 per cs cancer). Combining modalities, mpMRI or PSMA-PET, maximized detection (22 cs cancers, missing only 4), at an intermediate cost (EUR 81.991 total; EUR 3.727 per csPCa), while PSAD + mpMRI was the most parsimonious strategy, requiring only 20 biopsies but detecting just 12 cs cancers, thus missing 14 cases despite achieving the lowest per-detection cost (EUR 2.943 per cs cancer).

**Figure 2:**
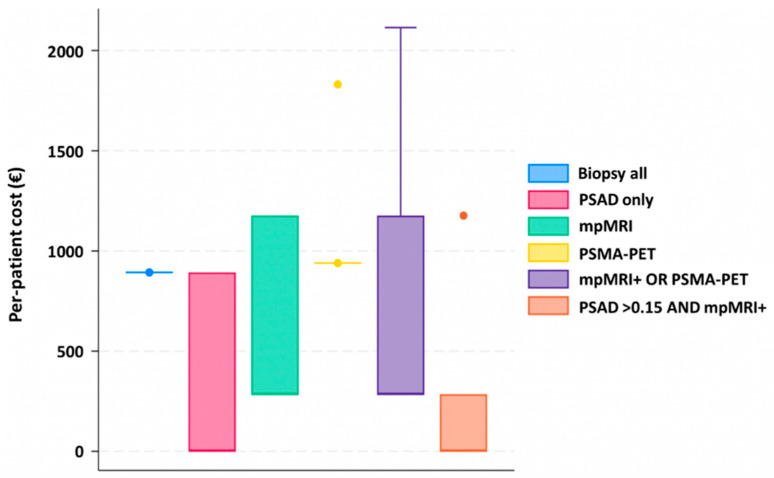
Distribution of costs per strategy.

Table 3 reports the pairwise comparison of each strategy vs. mpMRI alone (PI-RADS ≥ 3), which served as the reference. PSMA-PET: ICER was extremely high (~EUR 77.000 per additional csPCa detected), and INB remained negative across all WTP thresholds, indicating that PSMA-PET alone is not cost-effective. On the other hand, mpMRI or PSMA-PET: ICER was low (~EUR 2.900/extra csPCa), with INB consistently positive and increasing with higher WTP (EUR 80 at 5k, EUR 657 at 20k); therefore, this combination provided the most favorable cost-effectiveness profile. The updated ICER and INB with 95% CIs are shown in Table 4. Overall, combining mpMRI with PSMA-PET (mpMRI OR PET) represented the most cost-effective diagnostic pathway, balancing detection, efficiency, and cost. Figure 2 reports the graphical distribution of costs per triage strategy used for the analyses.

### 3.2. Comparison with Other Data in the Literature

In Table 5, the cost analysis studies for PSMA ligands for primary diagnosis of PCa are reported [14,15,16,17,18,19], for a total number of four studies, with three studies using [^68^Ga]PSMA-11 radiopharmaceutical and one using [^18^F]-PSMA-1007. Three papers analyzed cost-effectiveness prospectively, whereas one was retrospective. The best fit for primary diagnosis was reported by Privé et al. [19], comparing PSMA-PET with mpMRI. Therein, the authors report that in men with a negative mpMRI, adding a PSMA-PET does not seem to be cost-effective. In the other cases, the comparison was made with conventional imaging (i.e., CT and bone scan) in PCa staging. While all of these later papers acknowledged the higher diagnostic accuracy, two out of three also had lower costs but smaller QALY, whereas the retrospective one shows added costs to conventional imaging.

## 4. Discussion

**Figure 3:**
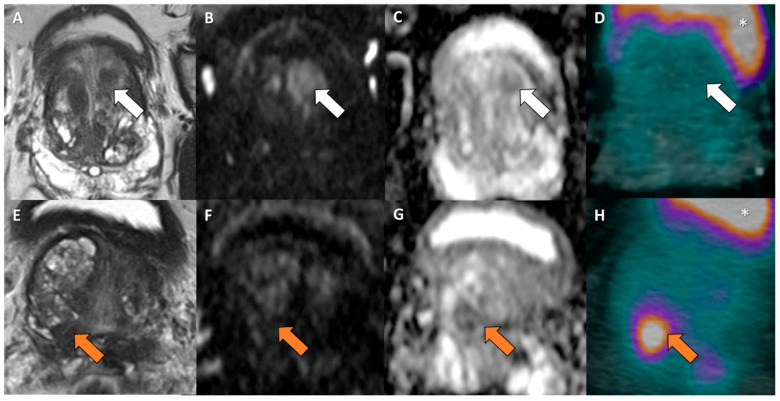
Comparison of two examples with extreme pathology results: (**A**–**D**) mpMRI-positive (PI-RADS 5) and PSMA-negative (PRIMARY score 1) of a patient with negative biopsy; (**E**–**H**) mpMRI-negative (PI-RADS 3) and PSMA-positive (PRIMARY score 5) of a patient with ISUP 5 (GS 9) adenocarcinoma of the prostate; (**A**,**E**) T2-weighted image with prostate; (**B**,**F**) diffusion-weighted imaging (DWI); (**C**,**G**) apparent diffusion coefficient (ADC); (**D**,**H**) axial fused [^68^Ga]PSMA-11 PET/CT (* defines the uptake in the bladder; arrows highlight the target lesions). Abbreviations: mpMRI = multiparametric magnetic resonance imaging; PI-RADS = Prostate Imaging–Reporting and Data System; ISUP = International Society of Urological Pathology; GS = Gleasons score.

The cost-effectiveness of PSMA-PET compared to mpMRI in PCa detection remains an evolving field with nuanced results. Current evidence indicates that mpMRI continues to be a highly cost-effective imaging approach for prostate cancer imaging, mainly due to its ability to reduce unnecessary biopsies through precise lesion detection and risk stratification. mpMRI offers advantages including widespread availability, relatively low cost, and well-established diagnostic performance [20]. Its high sensitivity and specificity position it as a reliable first-line modality in suspected PCa cases (Figure 3), directly lowering healthcare costs and patient morbidity associated with invasive procedures.

Incorporating PSMA-PET into diagnostic pathways—particularly for patients with equivocal or intermediate-risk mpMRI findings (e.g., PI-RADS 3 lesions)—demonstrates some cost-effectiveness potential, though it remains borderline. PSMA-PET enhances cancer detection [21], may avoid certain unnecessary biopsies, and reduces overdiagnosis of indolent low-risk tumors, potentially improving patient quality-adjusted life years (QALYs) [19]. However, PSMA-PET is substantially more expensive, requires specialized equipment and expertise, and has limited availability relative to mpMRI. Modeling studies show increased per-patient costs (approximately EUR 170-EUR 186 in equivocal cases), with incremental cost-effectiveness ratios (ICERs) in the moderately high range (EUR 56,700-EUR 93,212 per QALY gained). Thus, PSMA-PET’s cost-effectiveness in this setting depends on price reductions or careful patient selection to offset higher costs against clinical gains. Conversely, adding PSMA-PET after negative or low-risk MRI findings (i.e., PI-RADS 1–2) is generally not cost-effective, as it often leads to unnecessary biopsies prompted by additional PSMA-avid lesions without meaningful improvements in csPCa detection, thereby increasing costs and risks of overdiagnosis and overtreatment [19].

In the present study, we confirm that PSMA-PET alone lacks cost-effectiveness. However, combining mpMRI with PSMA-PET was the most favorable diagnostic pathway, effectively balancing detection rates, efficiency, and costs. On the other hand, PSAD + mpMRI pathway was the most resource-efficient strategy with the lowest cost per csPCa detection (EUR 2.943 per cs cancer), but it missed 14 csPCa cases (53.4%).

Our findings contrast with Privé et al. [19], who deemed PSMA-PET non-cost-effective post-negative mpMRI in primary biopsy settings (ICER EUR 52,300/QALY). This discrepancy arises from our re-biopsy population’s higher pre-test probability (csPCa 20% vs. 12%), enabling PSMA-PET’s high specificity (91% for PRIMARY ≥ 4) to rescue mpMRI false negatives efficiently. Sequential “OR” triage minimized biopsies (42/130) while detecting 85% csPCa at EUR 3727/case, versus standalone PSMA-PET’s unfavorable EUR 8023/case. European tariffs and short-term detection endpoints further favor combination in high-risk cohorts.

The choice of the PSMA ligand for PET/CT can significantly impact cost-effectiveness for primary prostate cancer diagnosis through differences in production, half-life, imaging quality, and operational efficiency [22]. [^68^Ga]PSMA-11 relies on short half-life nuclide (68 min) requiring on-site cyclotron or generator systems. This leads to high per-dose costs due to limited shelf life and frequent generator replacement. On the other side, fluorine-18 (110 min half-life) labeled tracers enable centralized production and distribution from commercial cyclotrons [22], leading to lower per-scan cost and wider availability and thus reducing infrastructure needs. Yet, [^68^Ga]PSMA-11 gives fewer false positives results, is more widely studied, presents an established high PPV (92%), and, in the re-biopsy setting, as proven in the PROSPET-BX trial [8,13], in combination with mpMRI, maximizes cost savings by identifying true negatives (95.5% avoidance rate in dual modality).

We acknowledge that our study presents some limitations. Firstly, based on the design and the total duration of the PROSPET-BX trial [8], we could not make a proper estimate of the clinical outcomes of the cohort and the corresponding cost-effectiveness of the strategies analyzed. Additionally, although the number of patients enrolled (*n* = 130) is adequate for the comparison of the diagnostic performance of PSMA-PET and mpMRI, it might be considered somehow limited for the purpose of an HTA (Health Technology Assessment) analysis. Third, the cost analyses in our study were based on the Italian National Health System. Consequently, further investigations are required to fully validate our findings on a larger, international scale.

To the best of our knowledge, this is the first study to prospectively perform cost analyses of PSMA-PET and mpMRI in the re-biopsy setting of patients with clinical and laboratory suspicion of PCa. Beyond re-biopsy triage, PSMA-PET/CT and mpMRI play complementary, indispensable roles throughout PCa management [15,23,24,25,26,27,28,29,30,31,32]. At primary diagnosis, mpMRI identifies index lesions, while PSMA-PET refines staging for pelvic nodes and metastases, enabling risk-adapted therapy. In metastatic settings, PSMA-PET outperforms conventional imaging for M1a/b detection, guiding PSMA-theranostics. Post-intervention monitoring benefits from sequential use: mpMRI evaluates local persistence, while PSMA-PET detects early recurrence or progression at distant sites. Our findings underscore this synergy in high-risk cohorts, supporting integrated protocols to optimize detection, treatment selection, and surveillance.

## 5. Conclusions

PSMA-PET and mpMRI play critical roles in detecting csPCa, but their economic impact and clinical benefits vary depending on the context of their use. In the present study, we investigated the cost-effectiveness of the aforementioned modalities within the PROPET-BX trial [8]. Our findings—the first produced for the re-biopsy setting—demonstrate that the combined strategy of “mpMRI or PSMA-PET” is the most cost-effective diagnostic pathway for csPCa detection.

## Figures and Tables

**Table 1:** Summary of specific costs per single procedure.

Procedure	Number	Single Cost (EUR)	Total Cost (EUR)
PSA	3 *	1.55	4.65
mpMRI	1	283.5	283.5
PSMA-PET	1	938	938
Fusion biopsy	1	809.4	809.4
Pathology	1	81.8	81.8
TOTAL			2117.35

Notes: * PSA, or prostate-specific antigen, is counted thrice based on PROSPET-BX protocol requirements for patient enrolment [8].

**Table 2:** Cost-effective analyses of the triage strategies.

Strategy	Biopsies	csPCa	NNT (Biopsies/cs)	Biopsies Avoided vs. All	csPCa Missed	Total Cost (EUR)	Cost per csPCa (EUR)
**Biopsy-all**	130	26	5.0	0	0	116.058	4464
**PSAD > 0.15**	61	17	3.6	69	9	54.565	3.210
**mpMRI+ (PI-RADS ≥ 3)**	34	17	2.0	96	9	67.357	3.962
**PSMA-PET+**	25	18	1.4	105	8	144.422	8.023
**mpMRI+ OR PET+**	42	22	1.9	88	4	81.991	3.727
**PSAD > 0.15 AND mpMRI+**	20	12	1.7	110	14	35.319	2.943

**Table 3:** Pairwise comparison of each strategy vs. mpMRI alone (PI-RADS ≥ 3).

Strategy	ICER (EUR/Extra csPCa)	INB WTP = 5.000	INB WTP = 10.000	INB WTP = 20.000
**PSMA-PET**	77.064	−554	−516	−439
**mpMRI OR PSMA-PET**	2.927	+80	+272	+657
**PSAD > 0.15**	—(no Δ effect)	+98	+98	+98
**PSAD > 0.15 AND mpMRI**	6.408	+54	−138	−523
**Biopsy-all**	5.411	−28	+318	+1.010

**Table 4:** Updated ICER and INB with 95% CIs.

Strategy	ΔcsPCa	ΔCost (EUR)	ICER (EUR/csPCa)	95% CI ICER (EUR)	INB EUR 5k (95% CI)	INB EUR 10k (95% CI)
**PSMA-PET**	+1	+77,065	77,065	(45,200; ∞)	−554 (−1120; −12)	−516 (−980; +28)
**mpMRI OR PSMA-PET**	+5	+14,634	2927	(1620; 8950)	+80 (22; 138)	+272 (198; 346)
**PSAD > 0.15**	0	−12,792	Dominated	N/A	+98 (62; 134)	+98 (62; 134)
**PSAD + mpMRI**	−5	−32,038	N/A (less eff.)	N/A	+54 (−112; 220)	−138 (−368; 92)
**Biopsy-all**	+9	+48,701	5411	(3450; 9820)	−28 (−162; 106)	+318 (162; 474)

**Table 5:** Summary of the cost analysis studies on PSMA ligands for primary diagnosis and staging of PCa.

Author, Year	Trial, Design	Cohort(s)	Tracer	Indication	Comparison	Findings
**de Feria Cardet et al. 2021** [14]	proPSMA [15], prospective	150 + 145	[^68^Ga]PSMA-11	High-risk PCa staging	CT and bone scan	PSMA PET/CT has lower direct comparative costs and greater accuracy compared to CI
**van der Sar et al. 2022** [16]	PSMA-PreRP trial [17], prospective	103	[^68^Ga]PSMA-11	Primary staging	CT, MRI, and bone scan	[^68^Ga]PSMA-11 PET/CT saves costs but results in small QALY loss
**Szczesniewski et al. 2024** [18]	Retrospective	100	[^68^Ga]PSMA-11	High-risk PCa staging	CT and bone scan; Choline PET	PSMA PET was the most accurate diagnostic option; the CI diagnostic workup was the most economical, and CI + PSMA was the most expensive
**Privé et al. 2025** [19]	Prospective	75 + 291	[^18^F]-PSMA-1007	Detection of csPCa	mpMRI	In men with a negative MRI, adding a PSMA-PET/CT does not seem to be cost-effective

## Data Availability

Data are available at https://doi.org/10.5281/zenodo.18825521 (accessed on 1 March 2025).
